# Correlation of ^18^F-Fluorodeoxyglucose Positron Emission Tomography Parameters with Patterns of Disease Progression in Locally Advanced Pancreatic Cancer after Definitive Chemoradiotherapy

**DOI:** 10.1016/j.clon.2017.01.038

**Published:** 2017-06

**Authors:** J.M. Wilson, S. Mukherjee, T.B. Brunner, M. Partridge, M.A. Hawkins

**Affiliations:** ∗ CRUK/MRC Oxford Institute for Radiation Oncology, Gray Laboratories, Oxford, UK; † Department of Radiation Oncology, University Hospitals Freiburg, Freiburg im Breisgau, Germany

**Keywords:** Chemoradiotherapy, FDG-PET, pancreatic cancer, treatment strategy selection

## Abstract

**Aims:**

A proportion of patients with pancreatic cancer never develop metastatic disease. We evaluated a role for ^18^F-fluorodeoxyglucose positron emission tomography (FDG-PET) in identifying a subset of patients with locally advanced pancreatic cancer (LAPC) who never develop metastatic disease and only experience local disease and may therefore benefit from local treatment intensification.

**Material and methods:**

Patients with histologically confirmed LAPC entered a single-centre phase II study of definitive upfront chemoradiotherapy (CRT). All patients underwent FDG-PET/CT before and 6 weeks after CRT. Tumour volume, standardised uptake values (SUV_max_, SUV_peak_, SUV_mean_, SUV_median_) and total lesion glycolysis (TLG) were measured on each scan and the response in each parameter was evaluated. The presence or absence of metastatic disease was noted on contrast-enhanced CT carried out every 3 months for 1 year and then at clinician discretion.

**Results:**

Twenty-three patients with LAPC were recruited; 17/23 completed treatment and had interpretable sequential imaging. Twenty-four per cent of patients only ever experienced local disease. Median pre-CRT FDG-PET parameters were significantly lower in patients with local disease only during follow-up compared with those who developed metastatic disease: SUV_max_ 3.8 versus 8.6 (*P* = 0.006), SUV_peak_ 2.5 versus 7.5 (*P* = 0.002), SUV_mean_ 1.8 versus 3.3 (*P* = 0.001), SUV_median_ 1.7 versus 3.0 (*P* = 0.002), TLG 26.9 versus 115.9 (*P* = 0.006). Tumour volume, post-CRT FDG-PET values and their relative change were not statistically different between local disease and metastatic disease groups. Receiver operating characteristic curves for pre-CRT FDG-PET parameters to predict those who never develop metastatic disease all had areas under the curve (AUCs) ≥ 0.932. Pre-CRT FDG-PET SUV_max_ < 6.2 predicted patients with local disease only during follow-up with 100.0% sensitivity and 92.3% specificity, 80.0% positive predictive value and 100% negative predictive value.

**Conclusions:**

Our findings suggest that patients with less FDG-avid tumours are less likely to metastasise and may therefore benefit from upfront local treatment intensification.

## Highlights

• A proportion of patients with locally advanced pancreatic cancer do not develop detectable metastatic disease.
• Locally advanced pancreatic tumours that are not FDG-avid tend not to metastasise and patients die because of locally progressive disease.
• This subgroup of patients may benefit from treatment interventions that aim to optimise local control.
• FDG-PET SUV_max_ offers a good summary of the metabolic activity of pancreatic tumours.

## Introduction

Pancreatic cancer has a poor prognosis and it is one of the few cancers that has not seen an improvement in its mortality rate in the last decade [1]. At diagnosis, 30% of patients have locally advanced pancreatic cancer (LAPC) [2]. Locally advanced tumours have not spread to distant organs, but cannot be surgically excised because of proximity to, or involvement of, key anatomical structures. It is known that a subgroup of pancreatic adenocarcinomas never metastasise. About 30% of patients therefore experience isolated locally progressive disease [3]. Despite this, the role of chemoradiotherapy (CRT) in LAPC remains controversial.

In a recent randomised phase III study (LAP07), patients who did not have disease progression after 4 months of chemotherapy (60%) were randomised to CRT or the continuation of chemotherapy. CRT did not lead to an improved overall survival compared with chemotherapy alone [4], although significantly less local tumour progression was seen in the CRT arm compared with the chemotherapy arm (34% versus 65%, *P* < 0.0001) [5]. Conversely, an Eastern Cooperative Oncology Group (ECOG) study that randomised patients with LAPC to gemcitabine alone or in combination with radiotherapy (50.4 Gy in 28 fractions) revealed a small, but statistically significant, improvement in overall survival with CRT compared with gemcitabine alone (11.1 versus 9.5 months, *P* = 0.017) [6]. The sequencing of treatment for patients with LAPC remains controversial. Although chemotherapy remains the mainstay of treatment, CRT has a role in a subset of patients and may improve local control and delay the onset of additional treatments [7]. A reliable means of identifying patients who do not develop metastatic disease may facilitate treatment strategy selection with a greater importance being placed on local treatment intensification with CRT in these patients. Loss of the tumour suppressor gene Smad4 (dpc4) is associated with the development of metastatic disease in pancreatic cancer [3], [8]. Although stratification of patients based on Smad4 (dpc4) is included in the currently recruiting Radiation Therapy Oncology Group (RTOG) 1201 study, the diagnosis of pancreatic cancer is often made on scant biopsy material or cytology, which limits the accuracy of SMAD4 immunohistochemistry [8]. A non-invasive method of predicting the pattern of disease progression *a priori* may aid treatment strategy selection on an individual patient basis.

^18^F-fluorodeoxyglucose positron emission tomography (FDG-PET) seems to have a prognostic role in pancreatic cancer [9]. In one large series of 260 patients with LAPC, a decline in maximum standardised uptake value (SUV_max_) of > 60% from pre- to post-CRT was associated with longer overall survival (41.9 versus 16.0 months) [10]. The usefulness of FDG-PET in predicting patterns of pancreatic cancer disease progression is not yet known. Therefore, the aim of this study was to look for correlations between FDG-PET parameters and patterns of disease progression to assess their usefulness in identifying patients with LAPC who will only ever have local disease and may therefore benefit from local treatment intensification. As SUV_max_, a single pixel value within a region of interest (ROI), is subject to considerable noise [11], a secondary aim was to compare SUV_max_ with other FDG-PET parameters to lend support to its ongoing use in pancreatic cancer as a summary of tumour FDG activity for use in studies to predict patterns of disease progression.

## Materials and Methods

### Patients

Patients with histologically confirmed, locally advanced pancreatic adenocarcinoma were enrolled in a single-centre phase II study (ARC2 clinical trial, EudraCT number 2008-006302-4). All patients gave written informed consent and the trial was approved by a regional ethics committee (REC No. 09/H0604/36). The clinical trial was carried out in accordance with the Code of Ethics of the World Medical Association (Declaration of Helsinki) for experiments involving humans. Patients who were found to have metastatic disease on the pre-treatment FDG-PET did not go on to receive CRT within the study and were therefore excluded from subsequent analysis. No induction chemotherapy was given to patients before the definitive CRT described below.

### Chemoradiotherapy Schedule

The treatment schedule has been described elsewhere [12]. In short, 50.4 Gy in 28 fractions was delivered to the primary pancreatic tumour and elective regional lymph nodes with a sequential boost of 9 Gy in five fractions to the gross tumour volume (GTV). Gemcitabine (300 mg/m^2^) and cisplatin 30 mg/m^2^ were given on weeks 1, 2, 4 and 5 of radiotherapy. In addition, nelfinavir was given at a dose of 1250 mg twice daily from 3 days before until the last day of CRT. Nelfinavir was started 10 days before radiotherapy until the last day of CRT following a protocol amendment (*n* = 6).

### Contrast-enhanced Computed Tomography

After fasting for 2 h, patients received 50 ml water orally just before contrasted-enhanced CT (CECT). Patients were imaged supine on a flat couch with knee rests, aligned using skin tattoos to a wall-mounted laser system, with arms above the head with a head support. Patients were scanned from above the dome of the diaphragm to the bottom of L4. A CT slice thickness of 2.5 mm was used.

### ^18^F-fluorodeoxyglucose Positron Emission Tomography/Computed Tomography Acquisition

**Fig 1:**
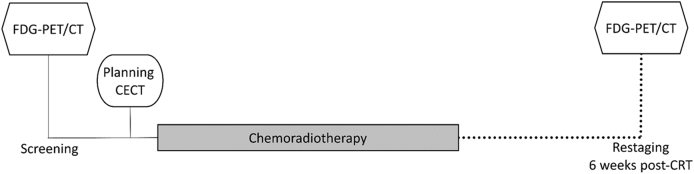
Timing of ^18^F-fluorodeoxyglucose positron emission tomography/computed tomography (FDG-PET/CT) in the ARC2 clinic trial.

FDG-PET/CT was carried out at baseline and 6 weeks after completing CRT. The imaging schedule is outlined in Figure 1. All scans were carried out on a GE Discovery 690. After fasting for 6 h and ensuring that the blood glucose was <10 mmol/l, FDG was injected at a dose of 4 MBq/kg (up to 600 MBq). PET acquisition was started after an uptake time of 90 min. Patients were scanned immobilised in the radiotherapy treatment position outlined above to aid accurate image co-registration to the CECT. The whole body from below the eyes to the mid-femurs was scanned. Scans were carried out in three-dimensions with a scan time of 4 min at each bed position. For the CT phase, 120 kV automA (maximum 250 mA), noise index 25.0 0.5 s/rotation, pitch 0.984:1, 3.75 mm slice width was used. Attenuation-corrected PET images were used in the analysis.

### Defining a Region of Interest for ^18^F-fluorodeoxyglucose Uptake Quantification

The CECT was loaded into Eclipse (version 13.0, Varian Medical Systems, Palo Alto, CA, USA) and the GTV was delineated by consensus of two radiation oncologists. The CECT was then rigidly registered to the CT component of the pre-CRT FDG-PET, prioritising accurate soft-tissue matching in the region of the GTV. The GTV was propagated on to the CT component of the FDG-PET/CT to generate a ‘tumour’ ROI. As the PET and CT components of the PET/CT share the same frame of reference, the ROI could then be copied to the PET image. The same process was carried out on the post-CRT FDG-PET/CT.

Images were visually assessed to ensure that the FDG uptake could be attributed to tumour FDG retention. Any areas of uptake extending beyond the GTV were noted and an explanation of the FDG-avidity was sought from the clinical information and imaging. As some reduction in tumour size could be seen on the CT component of the post-CRT FDG-PET/CT, some modification of the ROI was allowed on the post-CRT imaging.

The PET image was then imported into PMOD (Version 3.6, PMOD Industries, Zurich, Switzerland). FDG SUV_max_, SUV_peak_ (the 1 cm^3^ volume within the ROI with the highest mean activity), SUV_mean_, SUV_median_ and total lesion glycolysis (TLG = SUV_mean_ × tumour volume) were recorded for each image set. The percentage change in any parameter (%Δ) from pre- to post-CRT was calculated as follows:

%Δ = [(Parameter_post_ – Parameter_pre_)/Parameter_pre_] * 100

### Patient Follow-up

All patients had CECT of the chest, abdomen and pelvis every 3 months for 1 year. After 1 year, re-staging CTs were carried out at the discretion of the treating clinician. The timing and site of progression was noted. Imaging after the time of initial progression was also reviewed. For those who first progressed locally, subsequent imaging was reviewed and the presence or absence of metastatic disease following local progression was noted. Local progression was defined as progression at the site of primary disease and RECIST 1.1 criteria were used. Patient follow-up was until the time of death or, for three surviving patients, until censoring on 28 January 2015.

### Statistical Analysis

Data were analysed in SPSS Statistics 22 (IBM, Portsmouth, UK). Differences between groups were considered to be significant when *P* < 0.05. FDG-PET parameters were compared before and after CRT using the Wilcoxon signed ranks test. The correlation of SUV_max_ with the other PET-derived parameters at baseline and post-CRT was characterised by Spearman’s rho correlation coefficient and calculation of the *P* value of the correlation. Comparison of FDG-PET parameters was then carried out between patients with local disease only during follow-up and those who developed metastatic disease. Differences between these groups were sought by applying the Mann–Whitney *U* test. A receiver operating characteristic (ROC) analysis was carried out to define the optimal cut-off of FDG-PET-derived parameters in predicting patients who only ever had local disease during follow-up. The optimal cut-off was defined by the point on the ROC curve that minimised the number of false positives and maintained the identification of true positives.

## Results

### Patient Population

Between February 2010 and July 2014, 35 patients were screened and 23 patients were recruited. Seventeen per cent of screened patients were excluded from study entry because of metastatic disease on baseline FDG-PET. Of the 23 patients recruited to the study, four were not included in this subsequent analysis. One patient died of pneumonia less than 2 months after trial entry. Three patients did not complete treatment (one biliary sepsis, one fatal pulmonary embolism, one stroke). This left 19 patients with sequential FDG-PETs for analysis. One patient was then excluded because of diffuse FDG uptake around a common bile duct stent that was continuous with, and could not be differentiated from, the pancreatic tumour. Another patient was excluded because of poorly controlled blood glucose (>20 mmol/l) at the time of scanning, making image interpretation impossible. This left 17 patients (nine men, eight women, mean age 65 years, range: 43–74) with sequential imaging that could be used in this analysis.

### Timing of Investigations

The pre-CRT FDG-PET was carried out a mean of 22 days before study entry (range 14–34 days). The post-CRT was a mean of 6.0 weeks (range 5.4–7.0 weeks) after the last day of CRT.

### Changes in ^18^F-fluorodeoxyglucose Positron Emission Tomography Parameters

**Fig 2:**
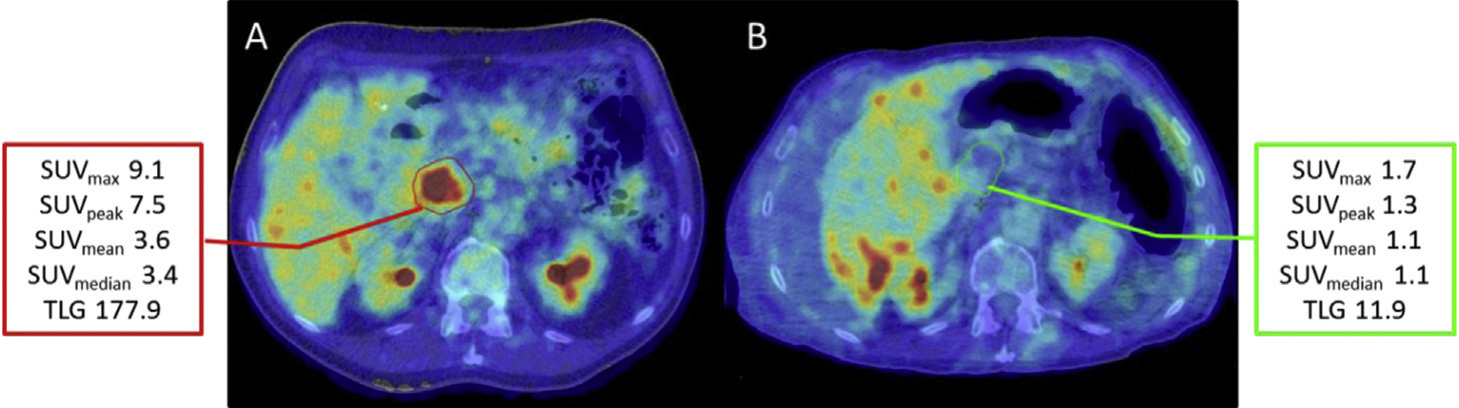
Transaxial images from pre- and post-chemoradiotherapy (CRT) ^18^F-fluorodeoxyglucose positron emission tomography/computed tomography (FDG-PET/CT) in a patient from the ARC2 study. Considerable changes can be seen from pre-CRT (A) and post-CRT (B). FDG avidity has decreased with a reduction in all of the PET parameters. The pre-CRT tumour region of interest (red) had to be modified on the post-CRT image (green). This is because the patient had developed ascites, which displaced the tumour and the liver. In addition, there was evidence of tumour shrinkage on the CT component of the PET/CT.

Changes in FDG-PET parameters from pre- to post-CRT are summarised in Table 1. There was a significant reduction in SUV_max_, SUV_peak_, SUV_mean_, SUV_median_ and TLG from pre- to post-CRT. A representative image of how FDG uptake changed from pre- to post-CRT can be seen in Figure 2. (Changes in the patient anatomy displayed here show the importance of accurate ROI definition for FDG uptake quantification.) All but one patient had a reduction in each of these values. One patient showed an increase in FDG uptake parameters.

### Identifying Patients Who Do Not Develop Metastatic Disease During Follow-up

From the 17 patients included in this analysis, 13 patients developed metastatic disease during follow-up. Four patients (24%) only ever experienced local disease. Each of these four patients showed evidence of local disease progression during the follow-up period. A statistically significant difference was observed in the pre-CRT FDG-PET parameters between those who had local disease only and those who went on to develop metastatic disease. Pre-CRT FDG-PET parameters that averaged uptake activity across the whole tumour ROI were clearly distinct between the two groups. TLG, which combines SUV_mean_ with tumour volume, weakened the correlation between the two groups that had been observed with SUV_mean_ alone.

**Fig 3:**
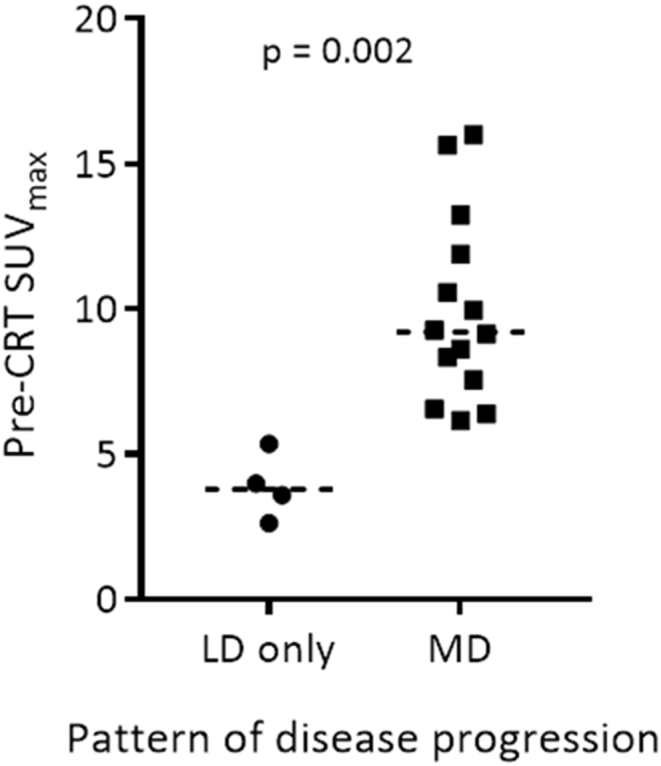
Pre-chemoradiotherapy maximum standardised uptake value (SUV_max_) in patients who experience only local disease and those who develop metastatic disease during follow-up.

The median baseline tumour volume, pre- and post-CRT FDG-PET parameters for patients in the local disease only group and those who developed metastatic disease can be seen in Table 2. The median pre-CRT SUV_max_ was 3.8 in those who had local disease only during follow-up compared with 8.6 in those who developed metastatic disease (*P* = 0.002), as seen in Figure 3.

There was no difference in pre-CRT tumour volume between these groups. Apart from post-CRT SUV_max_, there was no significant difference in the post-CRT SUVs or changes in SUV pre- and post-CRT between the local disease and metastatic disease groups.

A multivariate analysis was not carried out as the local disease only group only contained four patients. A predictive model would be unlikely to correctly identify only four of 17 patients.

### Receiver Operating Characteristic Curve Analysis for Identifying Patients with Only Local Disease

A ROC curve analysis for all of the pre-CRT FDG-PET parameters can be seen in Table 3, showing pre-CRT SUV_mean_ to be the best predictor of those who never experience metastatic disease. The more easily accessible parameter, SUV_max_, performed well when quantified pre-CRT (AUC = 0.932). The pre-CRT parameters seem to be more predictive of patients who only ever have local disease rather than progressing distally than post-CRT parameters.

### Maximum Standardised Uptake Value (SUV_max_) Correlates Closely with Other ^18^F-fluorodeoxyglucose Positron Emission Tomography Parameters in Locally Advanced Pancreatic Cancer

**Fig 4:**
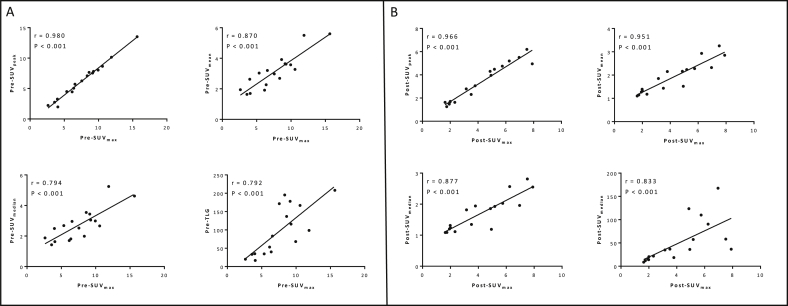
Scatterplots showing the correlation of maximum standardised uptake value (SUV_max_) ^18^F-fluorodeoxyglucose positron emission tomography (FDG-PET) parameters both before chemoradiotherapy (A) and after chemoradiotherapy (B) with other FDG-PET-derived parameters.

Correlation of pre-CRT SUV_max_ with SUV_peak_, SUV_mean_, SUV_median_ and TLG can be seen in Figure 4A. The closest correlation can be seen between pre-CRT SUV_max_ and SUV_peak_ (Spearman’s rho = 0.980), although all parameters have a significant correlation with SUV_max_ despite the parameter values being significantly different (*P* < 0.001). Spearman’s rho for the correlation of SUV_max_ with SUV_mean_, SUV_median_ and TLG was 0.870, 0.794 and 0.792, respectively (*P* < 0.001 for all parameter correlations) pre-CRT.

A very similar pattern was seen in the post-CRT FDG-PET images (see Figure 4B), with the closest correlation being observed between SUV_max_ and SUV_peak_. Spearman’s rho for the correlation of SUV_max_ with SUV_peak_, SUV_mean_, SUV_median_ and TLG was 0.966, 0.951, 0.877 and 0.833, respectively (*P* < 0.001 for all parameter correlations) post-CRT. The weakest, yet still significant, correlation was seen between SUV_max_ and TLG at both time points.

## Discussion

LAPC has a poor prognosis and recent evidence has suggested that CRT does not improve survival compared with chemotherapy alone [4]. As we know that some patients only ever experience disease at the site of the pancreatic tumour, it would seem that there should be a role for upfront locally intensive treatment in a subgroup of patients – if only this group could be accurately identified. Our findings suggest that the inclusion of FDG-PET at the point of diagnosis may help guide treatment strategy selection. Of note, 17% of patients screened for entry into this study (ARC2) were excluded because of the identification of previously unrecognised metastatic disease on the screening FDG-PET scan. Despite this, FDG-PET is not currently part of the routine staging investigations for LAPC in the UK. This may change following reporting of the PET-PANC study, which showed that FDG-PET influenced management decisions in patients with suspected pancreatic cancer in 45% of cases, including preventing futile resection in 20% of patients due to have surgery [13]. Our findings presented here suggest that patients with LAPC with tumours that have low FDG-avidity are less likely to develop metastatic disease. This patient group may therefore benefit from intensification of local treatment.

The derivation of FDG-PET/CT-derived parameters is not a process that can be automated and the imaging analysis should always take the clinical context and pattern of FDG uptake into account. Although it would have been possible to obtain parameter values for two patients who were excluded from this analysis (one because of elevated serum glucose at the time of scanning and one because of inflammation around a common bile duct stent), the activity that is being quantified would not have related to tumour glucose metabolism. The importance of accurate placement of an ROI by careful review of the anatomical information in the CT component of the PET/CT is highlighted in Figure 3. This observation also supports our decision to use a CECT-defined tumour ROI rather than an automated method based on FDG uptake.

Once patients who did not complete therapy and those with incomplete or uninterpretable imaging were excluded, only 17 patients were able to be included in this analysis. FDG-PET staging and response assessment is not currently routine in LAPC. Patients recruited to recently published series SCALOP and LAP07 did not routinely have FDG-PET studies [14], [15]. All of our observations and recommendations are therefore presented with a degree of caution as they are drawn from such a small sample.

The impact of the variability in timing of the imaging tests in relation to the start of CRT is uncertain. The mean time pre-CRT that the FDG-PET was carried out in this series was 22 days, with a range of 14–34 days. This variability should be taken into account when analysing a larger cohort. The potential for FDG-PET progression over 14–34 days in LAPC is not known, but could affect the predictive utility of the test.

The median value of all of the assessed FDG-PET parameters decreased after CRT. A marked heterogeneity in response was seen. For example, the %ΔSUV_max_ varied from an increase of 97.6% to a decrease of 80.9%. This heterogeneity may explain the usefulness of the %ΔSUV_max_ parameter in informing about patient prognosis in this cohort of patients. We have reported that patients with a reduction in SUV_max_ greater than the median for this cohort had a prolonged overall survival compared with those with a reduction in SUV_max_ less than the median (23.0 versus 14.6 months; *P* = 0.01) [12]. The role of FDG-PET response assessment requires further study.

Our finding that pre-CRT FDG-PET parameters tend to be lower in those patients who only experience local disease and did not develop metastatic disease during follow-up shows promise. Obtaining these values in a larger cohort of patients with LAPC treated with upfront CRT may identify which parameters are most useful in identifying patients who only ever have local disease. Our sample size, particularly as only four patients were in the local disease group, precluded a multivariate analysis to identify the most useful parameters. Our observation that patients with low pre-CRT FDG uptake (thresholds identified by ROC curve analysis: SUV_max_ < 6.2, SUV_peak_ < 4.5, SUV_mean_ < 2.1, SUV_median_ < 1.9) never seem to develop metastatic disease potentially identifies a patient group who may benefit from upfront CRT, potentially with dose escalation of the radiotherapy to achieve lasting local control. Previous attempts at radiotherapy dose escalation in LAPC have been limited by normal tissue toxicity – most notably to the duodenum [16]. Validation of our findings in another cohort of patients may support a clinical trial investigating isotoxic radiotherapy dose escalation in low FDG uptake tumours or a change in treatment sequencing with a priority given to CRT over intensification of systemic chemotherapy may be the next step in improving outcomes for a subset of patients with LAPC. There is a trend in recent studies in LAPC [14], [17] to offer induction chemotherapy followed by CRT in those who do not progress distally. Achieving good local control, early in the treatment schedule, may translate into a clinical benefit in patients in the local disease group. The best means of achieving lasting local control in this subset of patients with tumours that have low FDG-avidity is not clear. The application of conventionally fractionated CRT regimens and hypofractionated treatments, including stereotactic ablative radiotherapy, warrants further investigation. As chemotherapy remains the mainstay of therapy in LAPC [7], [18], repeating this study in patients who will receive chemotherapy as their first treatment may be of benefit – both to further explore the prognostic value of FDG-PET in these patients, but also as a means of directing clinical decisions.

It might have been expected that the post-CRT FDG-PET would be more informative about patterns of disease progression than the pre-CRT imaging. Protease inhibitors, like nelfinavir, induce peripheral insulin resistance and with long-term use can impair insulin secretion [19]. The effect this may have on FDG-PET parameters is not known. The post-CRT PET was carried out a mean of 6 weeks after CRT. This may be too early after finishing treatment for residual cancer cells to resume metabolic activity. Imaging too early after radiotherapy can lead to falsely elevated FDG-PET parameters because of ongoing treatment-related inflammation. The optimal time to perform FDG-PET after CRT for pancreatic cancer for both prognostication and to predict patterns of disease progression requires further investigation. The observation that the pre-CRT FDG-PET was more useful in identifying patients who did not go on to develop metastatic disease is the most useful result for clinical decision making. The tumours were also treatment naive, meaning there are few factors influencing the tumour glucose metabolism other than its intrinsic biology. It is, therefore, perhaps unsurprising that scanning at this time point was the most informative.

Although we have previously reported that the relative change in FDG-PET parameters from pre- to post-CRT offered prognostic information in this patient cohort [12], these parameters were not useful in predicting which patients would develop detectable metastatic disease and those who would not. This may be because of the low SUVs seen in the tumours of those who did not develop metastatic disease. The failure of the tumours to take up glucose pre-CRT means that a less marked change was seen after CRT. The biology behind the difference between glucose uptake and the pattern of disease progression requires clarification. It also points to the observation that mortality in LAPC can be due to local disease progression and not just distant failure, which further emphasises the need to find measures to optimise local control.

Although SUV_max_ correlates very closely with SUV_peak_, SUV_mean_ and SUV_median_, the correlation with TLG is significant, but weaker than with the other parameters. This is, perhaps, unsurprising given that TLG factors in tumour volume. It is known that pancreatic tumours have a large stromal component. The stroma, composed of fibroblasts, pancreatic stellate cells, immune cells and extracellular matrix proteins such as types I and III collagens [20], contributes to the volume of the tumour, but may in comparison with the tumour cells be relatively metabolically quiescent. This observation may also explain the failure of tumour volume alone to predict patients with only local disease when other pre-CRT variables differed in this group. It is not the size of pancreatic tumours that informs behaviour, but rather the metabolic activity within this volume.

The SUV_peak_ is the activity in a 1 cm^3^ sphere the highest mean activity within the ROI. It was thought that using the SUV_peak_ rather than SUV_max_ would reduce some of the uncertainty associated with the SUV_max_ because of the effect of noise on this single pixel parameter. The observed results suggest that SUV_max_ and SUV_peak_ correlate extremely well (Spearman’s rho = 0.980) in LAPC, despite the numerical values being significantly different. This is reassuring, as SUV_max_ is the most commonly used PET parameter in response prediction studies and is often routinely available in routine clinical practice. This finding needs to be replicated in a larger cohort of patients before the detailed analysis of uptake values across the whole ROI is no longer taken into account. The calculation of SUV_peak_, SUV_mean_ and SUV_median_ is more labour intensive than simply obtaining the maximum value, but seems to be worthwhile in other tumour sites [21], [22], [23]. Although this series is too small to show any real difference in the ability of the parameters to identify patients with only local disease, there is a suggestion that SUV_peak_, SUV_mean_ and SUV_median_ may be more robust than SUV_max_ and TLG given the smaller *P* values and greater ROC curve AUC.

FDG-PET/CT-derived parameters have been shown to be prognostic in pancreatic cancer in both surgical and CRT series [10], [24], [25]. To our knowledge, this is the first report of FDG-PET/CT being used to predict patterns of disease progression after definitive treatment. As pre-CRT FDG-PET/CT was most useful in identifying a subset of patients who did not develop metastatic disease during follow-up, it offers an *a priori* prediction of the probable pattern of disease progression that, if validated, could be used in treatment strategy selection.

## Conclusions

Pre-CRT FDG-PET parameters can identify patients who will probably only ever have cancer localised to the pancreas. These findings suggest that patients with less FDG-avid tumours are less likely to develop metastatic disease and may therefore benefit from upfront local treatment intensification.

The application of FDG-PET to treatment strategy selection in LAPC therefore shows promise, but these findings require testing and validation in a larger cohort.

SUV_max_ is easy to obtain from FDG-PET images and is the most commonly used parameter in response prediction studies. Although this single pixel value is subject to noise, it was significantly correlated with SUV_mean_, SUV_median_, SUV_peak_ and TLG in this cohort of patients with LAPC when assessed both before and after CRT. This observation supports the ongoing use of SUV_max_ in FDG-PET studies in LAPC.

## Figures and Tables

**Table 1:** Pre-chemoradiotherapy (CRT), post-CRT and relative change (%Δ) in ^18^F-fluorodeoxyglucose positron emission tomography (FDG-PET)-derived parameters

Parameter	Pre-CRT Median (range)	Post-CRT Median (range)	%Δ Median (range)	Significance *P* value (Wilcoxon signed ranks test)
SUV_max_	7.6 (2.6 to 15.6)	3.8 (1.6 to 7.9)	−44.2 (97.6 to −80.9)	0.002
SUV_peak_	6.3 (2.0 to 13.5)	3.1 (1.6 to 6.1)	−41.4 (51.3 to −83.3)	0.001
SUV_mean_	3.0 (1.6 to 5.6)	1.9 (1.1 to 3.3)	–35.1 (8.6 to –67.0)	<0.001
SUV_median_	2.7 (1.6 to 5.2)	1.8 (1.1 to 2.8)	−33.5 (2.4 to −68.3)	<0.001
TLG	83.3 (16.8 to 208.0)	36.0 (8.8 to 167.5)	−46.5 (4.0 to −93.3)	<0.001

SUV, standardised uptake value; TLG, total lesion glycolysis.

**Table 2:** Comparison of pre- and post-chemoradiotherapy (CRT) and relative change in ^18^F-fluorodeoxyglucose positron emission tomography (FDG-PET)-derived parameters in patients who never developed distant disease during follow-up (local disease only) and those did develop metastatic disease

Parameter	Local disease only during follow-up Median (range)	Developed distant disease during follow-up Median (range)	Significance *P* value (Mann-Whitney *U* test)
Baseline tumour volume (cm^3^)	21.6 (1.6 to 38.0)	32.9 (11.3 to 80.2)	0.202
Pre-SUV_max_	3.8 (2.6 to 6.2)	8.6 (4.0 to 15.6)	0.002
Pre-SUV_peak_	2.5 (2.0 to 4.5)	7.5 (4.5 to 13.5)	0.002
Pre-SUV_mean_	1.8 (1.6 to 1.9)	3.3 (2.2 to 5.6)	0.001
Pre-SUV_median_	1.7 (1.4 to 1.9)	3.0 (2.5 to 5.2)	0.002
Pre-TLG	26.9 (16.8 to 53.3)	115.9 (35.2 to 208.8)	0.006
Post-SUV_max_	2.0 (1.6 to 3.5)	4.9 (1.7 to 7.9)	0.045
Post-SUV_peak_	1.7 (1.6 to 2.3)	4.3 (1.3 to 6.2)	0.102
Post-SUV_mean_	1.4 (1.1 to 1.4)	2.2 (1.1 to 3.3)	0.060
Post-SUV_median_	1.3 (1.1 to 1.3)	1.9 (1.1 to 2.8)	0.130
Post-TLG	17.2 (8.8 to 36.7)	36.6 (15.1 to 167.5)	0.102
%ΔSUV_max_	−43.6 (−24.5 to −59.8)	−45.7 (97.5 to −80.9)	0.785
%ΔSUV_peak_	−31.7 (−17.8 to −48.1)	−45.2 (51.2 to −83.3)	0.384
%ΔSUV_mean_	−27.0 (−19.8 to −35.1)	−42.2 (8.6 to −67.0)	0.130
%ΔSUV_median_	−25.4 (−13.6 to −30.0)	−45.2 (51.3 to −83.3)	0.202
%ΔTLG (%)	−35.2 (−30.0 to −47.7)	−56.5 (4.0 to −93.0)	0.296

SUV, standardised uptake value; TLG, total lesion glycolysis.

**Table 3:** Receiver operating characteristic (ROC) curve analysis of the ability of pre-chemoradiotherapy ^18^F-fluorodeoxyglucose positron emission tomography (FDG-PET) to predict patients who will only ever have local disease (no metastatic spread)

Parameter	ROC curve AUC	95% confidence interval	Parameter cut-off	Sensitivity (%)	Specificity (%)	PPV (%)	NPV (%)
Pre-SUV_max_	0.932	0.802–1.000	6.2	100.0	92.3	80.0	100.0
Pre-SUV_peak_	0.977	0.909–1.000	4.5	100.0	92.3	80.0	100.0
Pre-SUV_mean_	1.000	1.000–1.000	2.1	100.0	100.0	100.0	100.0
Pre-SUV_median_	0.977	0.909–1.000	1.9	100.0	92.3	80.0	100.0
Pre-TLG	0.932	0.787–1.000	60.7	100.0	76.9	57.1	100.0

AUC, area under the curve; PPV, positive predictive value; NPV, negative predictive value; SUV, standardised uptake value; TLG, total lesion glycolysis.
